# Bacitracin Methylene Disalicylate Improves Intestinal Health by Modulating Its Development and Microbiota in Weaned Rabbits

**DOI:** 10.3389/fmicb.2021.579006

**Published:** 2021-06-25

**Authors:** Yang Chen, Shuaishuai Hu, Jiali Li, Bohao Zhao, Naisu Yang, Tong Zhou, Shuang Liang, Shaocheng Bai, Xinsheng Wu

**Affiliations:** ^1^College of Animal Science and Technology, Yangzhou University, Yangzhou, China; ^2^Joint International Research Laboratory of Agriculture and Agri-Product Safety, Yangzhou University, Yangzhou, China

**Keywords:** bacitracin methylene disalicylate, intestinal health, microbiome, weaned rabbits, peptidoglycan recognition protein

## Abstract

Intestinal infections are a major cause of morbidity and mortality in humans and agricultural animals, especially newborns and weaned animals. Preventive treatments that help weaned animals maintain homeostasis and balance the hindgut microbial populations are desirable. The present study aimed to explore the impact of bacitracin methylene disalicylate (BMD) on the intestinal health by analyzing the intestinal environment, morphology, expression of peptidoglycan recognition proteins (PGRPs), and flora of weaned rabbits. A total of 300 New Zealand weaned rabbits were randomly divided into the following five treatment groups for a 35-day feed trial: control group (basal diet), bacitracin zinc (BZ) group (50 mg/kg BZ), BMDa group (100 mg/kg BMD), BMDb group (50 mg/kg BMD), and BMDc group (rabbits fed a basal diet supplemented with 25 mg/kg BMD). In each treatment group, 28 rabbits were slaughtered for experimental analysis. The results showed that the supplementation of BMD increased the environmental acidity of the cecum of the weaned rabbits and reduced the ammonia-nitrogen concentration, which was beneficial to the survival of useful bacteria in the intestine. The morphology analysis of the duodenum using hematoxylin and eosin staining revealed that the villus length, villus/crypt ratio, and intestinal wall thickness increased in the BMD group, thereby improving the structure of the duodenum and the absorption capacity of the small intestine. Moreover, real-time polymerase chain reaction test showed that PGRPs (especially PGLYRP-1 and PGLYRP-2) in the intestinal had an antagonistic effect with BMD in the process of inhibiting pathogenic bacteria, resulting in their decreased expression (*P* < 0.05). Furthermore, through 16S rRNA sequencing in the cecal content, the abundance of the predominant phyla in the BMDa and BZ groups was found to be the closest. The abundance of the genera *Lachnospira*, *Erysipelotrichaceae (p-75-a5*), *Paraprevotellaceae* (*YRC22*), *Mogibacterium*, *Peptococcaceae* (*rc4-4*), *Anaerovibrio*, *Succinivibrio*, and *Sphaerochaeta* increased in the BMDa and BZ groups (*P* < 0.05). The relative abundance of *Alistipes*, *Sedimentibacter*, and *Dorea* significantly increased only in the BMDa group (*P* < 0.05). Conclusively, BMD, as well as microbes, improved the intestinal environment and structure to maintain the intestinal health of weaned rabbits.

## Introduction

Intestinal infections are a major cause of morbidity and mortality worldwide in humans and agricultural animals, especially newborns and weaned animals. The neonatal phase in humans and animals is one of the critical periods in life, during which nutrition and management have a long-term effect on the overall performance (Videhult and West, 2016). In this stage, they grow fast and have a strong metabolism; however, the digestive system is incompletely developed, and the intestinal flora balance is easily affected. The intestine contains a microbial ecosystem, and a balanced microbiome is important in the development of a healthy intestinal mucosal immune system (Laukoetter et al., 2008). Under normal circumstances, the intestinal flora and intestinal immunity are in a dynamic balance through precise control mechanisms. Once this mechanism is destroyed, a series of diseases ensue. Therefore, preventive treatments that help weaned rabbits maintain homeostasis and balance the gut microbial populations are desirable.

Bacitracin methylene disalicylate (BMD) is the reaction product of *Bacillus licheniformis* secondary metabolites, bacitracin and methylene salicylic acid. BMD has a strong inhibitory effect on Gram-positive bacteria. On the contrary, BMD has no inhibitory effect on intestinal probiotics, such as *Lactobacilli*, *Bifidobacteria*, and *Bacillus*. It has a promoting effect on the growth and intestinal health of livestock, and has been used in the breeding of broilers, pigs, and cattle (Damron and Wilson, 1985; Song et al., 2011; Xie et al., 2013). The role of BMD in intestinal flora balance in rabbits needs further investigation. Moreover, BMD can restore intestinal flora balance and control gastrointestinal infections in rabbits.

Besides the intestinal mucosal epithelial barrier and the intestinal flora, the intestinal health also depends on the immune barrier formed by intestinal immune cells and their secretions. Peptidoglycan recognition protein (PGRP), as a bactericidal protein in innate immunity, can maintain normal intestinal flora and is associated with inflammatory bowel disease (IBD). PGRP-Iα and Nod2 can cooperate to maintain the intestinal flora balance and resist colitis (Jing et al., 2014). Several non-catalytic PGRPs can act as selective peptidoglycan receptors in the upstream of Toll and IMD pathways in *Drosophila* (Zaidman-Remy et al., 2011). A previous study found that diarrhea in weaned rabbits led to the upregulation of *PGRPs* in the cecum and duodenum (Chen et al., 2017). Therefore, the effect of the interaction between BMD and PGRP on the homeostasis of intestinal microorganisms and intestinal immunity needs further analysis.

Further, a previous study found that the addition of BMD to the diet of weaned rabbits improved their growth performance and significantly reduced diarrhea (16.67 vs. 28.33%) and mortality (5 vs. 16.67%) rates (Hao et al., 2016). Based on these responses, this study aimed to further compare the effects of BMD in the diet on intestinal health by analyzing the intestinal environment, morphology, expression of intestinal PGRPs, and intestinal flora of weaned rabbits. The findings revealed the feasibility of using BMD to restore intestinal flora balance and control gastrointestinal infections.

## Materials and Methods

### Ethics Statement

This study was strictly performed following the regulations for experimental animals of the China Department of Agriculture and approved by the Institutional Animal Care and Use Committee of Yangzhou University (Jiangsu, China).

### Experimental Design and Animal Management

A total of 300 New Zealand weaned rabbits (35 days of age, 633.9 ± 32.62 g) were selected as experimental animals. The animals were randomly divided into the following five treatment groups (*n* = 60, including 30 male and 30 female) for a 35-day feed trial: (1) control group (rabbits fed a basal diet), (2) bacitracin zinc (BZ) group (rabbits fed a basal diet supplemented with 50 mg/kg BZ), (3) BMDa group (rabbits fed a basal diet supplemented with 100 mg/kg BMD), (4) BMDb group (rabbits fed a basal diet supplemented with 50 mg/kg BMD), and (5) BMDc group (rabbits fed a basal diet supplemented with 25 mg/kg BMD). The basal diet was formulated according to the nutrient requirements of weaned rabbits. The ingredient composition and nutrient levels are shown in Supplementary Table 1. This study used BZ, a polypeptide antibiotic, as the positive control. The feeding and management methods of rabbits in each group were the same. The feed was processed into pellets for free feeding and drinking. No vaccines or antibiotics were administered to these rabbits throughout the experiment.

### Sampling Procedure

The rabbits were euthanized, and the pH value of the cecal contents was immediately measured. Afterward, the cecal contents were taken in a 2-mL cryopreservation tube and stored in liquid nitrogen for detecting of volatile fatty acids (VFAs) and ammonia-nitrogen (NH_3_-N). Subsequently, the parts of the digestive tract were carefully separated to identify different segments of the intestines, and a 2- to 3-cm section of the intestine was cut from the same part of the duodenum. After cleaning the intestinal canal of the duodenum with 0.9% physiological saline, the canal was placed in a preformulated paraformaldehyde solution for fixation and used for preparing intestinal tissue sections. A 1- to 2-cm section of the intestinal canal was taken from the duodenum and cecum of the rabbits into a 2-mL cryopreservation tube and stored in liquid nitrogen for total RNA extraction. The samples were collected from 28 rabbits in each group, and 8 were used for microbial analysis.

### Determination of Intestinal Environmental Indicators

The contents of the cecum were immediately taken out, and the pH value was quickly determined using a DELTA 320 pH meter (Mettler-Toledo, Shanghai, China). Furthermore, 2 mL of distilled water was added to 2 mL of cecal contents and centrifuged at 10,000 rpm for 10 min. Then, 1 mL of supernatant was taken, 0.2 mL of 20% metaphosphoric acid solution containing 60 mM crotonic acid was added, and 0.4 μL of supernatant was taken for injection analysis. The VFA was measured using a GC-9A Gas Chromatograph (Shimadzu, Kyoto, Japan), with nitrogen as the carrier gas and a flow rate of 30 mL/min. The standard solutions were 3.353 mg/mL acetic acid, 1.189 mg/mL propionic acid, and 0.793 mg/mL butyric acid. In addition, after centrifuging 4 mL of cecal content diluent for 10 min, 50 μL of the supernatant was taken in a 10-mL test tube. Subsequently, 3 mL each of phenol and sodium hypochlorite was added to the test tube. The test tube was cooled in a 60°C water bath for 10 min and then immediately with cold water. Then, the optical density (OD) at 546 nm was determined using a UV756 spectrophotometer (UNICO, CA, United States) to calculate the NH_3_-N concentration in the cecum. The ammonia standard stock solution contained 32 mg/100 mL NH_3_-N.

### Determination of Intestinal Villus Height and Crypt Depth

The duodenal intestinal contents were cleaned with ice-cold physiological saline, and the intestinal canal of about 2–3 cm at the same location was collected and fixed in 4% paraformaldehyde solution. Subsequently, the fixed tissue was taken out, rinsed with running water, and then put into gradient alcohol for dehydration. The samples were cleared with xylene for about 15–20 min, dipped in wax, and embedded at 60°C. Next, after sectioning, patching, and dewaxing, hematoxylin–eosin was used for staining. The slices were observed using an optical microscope, photographed, and stored for later analysis. Each section of the intestinal tissue was cut continuously into five pieces, and six typical fields of view (clear pictures and complete villi) were selected from each tissue section. For each field of view, the longest villi and the corresponding crypt depth were selected for measurement. The average values of the measurements were recorded, and the villus height and crypt depth were calculated.

### Analysis of Intestinal Gene Expression of PGRPs

The total RNA of the intestinal tissue was extracted with TRIzol. The reverse transcription (RT) reaction system was prepared using a SuperRT cDNA Kit following the manufacturer’s protocols. The system included 2 μL of the RNA template, 4 μL of dNTP Mix (2.5 mmol/L), 2 μL of Prime Mix, 4 μL of 5 × RT buffer, 1 μL of SuperRT 200 U/μL, and RNase-free water to a volume of 20 μL. Real-time quantitative RT–polymerase chain reaction (qRT-PCR) was carried out using an Applied Biosystems 7500 Real-Time RT-PCR System (Applied Biosystems, CA, United States). The following program was used: 95°C for 30 s, followed by 40 cycles of 95°C for 5 s and 60°C for 34 s. According to the mRNA sequences of rabbit PGRPs published in GenBank, qRT-PCR primers of peptidoglycan recognition protein (*PGLYRP*)*-1*, *PGLYRP-2*, and *PGLYRP-3* were designed, and are shown in Supplementary Table 2. Each sample was measured in triplicate. The 2^–ΔΔCt^ method was used to quantitate and analyze the expression of target genes, and glyceraldehyde-3-phosphate dehydrogenase (*GAPDH*) was the reference gene.

### Microbial Analysis

The total DNA of the cecal contents was extracted using hexadecyl trimethyl ammonium bromide. Genomic DNA was amplified using the specific primers 515F 5′-GTGCCAGCMGCCGCGGTAA-3′ and 806R 5′-GGACTACHV GGGTWTCTAAT-3′ with barcode-specific primers in the V4 region of the bacterial *16S rRNA* gene. The PCR products were evaluated using a Qubit R 2.0 Fluorometer (Life Technologies, CA, United States) and an Agilent Bioanalyzer 2100 system (Agilent Technologies, CA, United States). The amplicon sequencing library was subsequently constructed, and Illumina HiSeq was used for *16S rRNA* gene sequencing.

According to the barcode and primer sequence, data of each sample were obtained from the off-machine sequence. After truncating the barcode and primer sequences, the reads of each sample were spliced using FLASH (version 1.2.7^1^) (Magoc and Salzberg, 2011). The Quantitative Insights Into Microbial Ecology (QIIME, version 1.7.0^2^) process was used for quality control and data processing (Caporaso et al., 2010). The effective tags of the samples were clustered using the UPARSE software (version 7.0.1001^3^), and the sequences were clustered into operational taxonomic units (OTUs) with >97% identity (Edgar, 2013). Meanwhile, the sequences with the highest frequency were selected as the representative sequences of OTUs according to their algorithm principles. Species annotations were analyzed using Ribosomal Database Project (RDP, version 2.2^4^) Classifier and Greengene databases^5^ (with a threshold of 0.8–1), and the community composition of each sample was counted at the kingdom, phylum, class, order, family, genus, and species levels (DeSantis et al., 2006; Wang et al., 2007). The data were normalized with the least amount of data as the standard. The alpha- and beta-diversity analyses were assessed on the data after normalization by in-house Perl scripts. The QIIME software was used to calculate the observed species, Chao1, Shannon, Simpson, and abundance-based coverage estimator (ACE) indices. The vegan package of R software (version 2.15.3) was used for non-metric multidimensional scaling (NMDS) analysis to reflect the differences between and within samples (Noval Rivas et al., 2013). The raw data for this project were deposited with the national center for biotechnology information (NCBI, accession number: PRJNA530911).

### Statistical Analysis

For microbial analysis, the multiresponse permutation procedure (MRPP) was used to analyze whether significant differences existed in microbial community structure. Metastat analysis was carried out using R software (version 2.15.3) to find the species with significant differences (White et al., 2009). At each classification level, permutation tests were performed to obtain the *P*-values, and the Benjamini and Hochberg false discovery rate method was used to further correct the *P*-values. Other indices except microbial analysis were used to evaluate the significance using the one-way analysis of variance of SPSS 21. The results were expressed as mean ± standard deviation in tables and figures at the level of significance (*P* < 0.05).

## Results

### Intestinal Morphology in Weaned Rabbits

**FIGURE 1:**
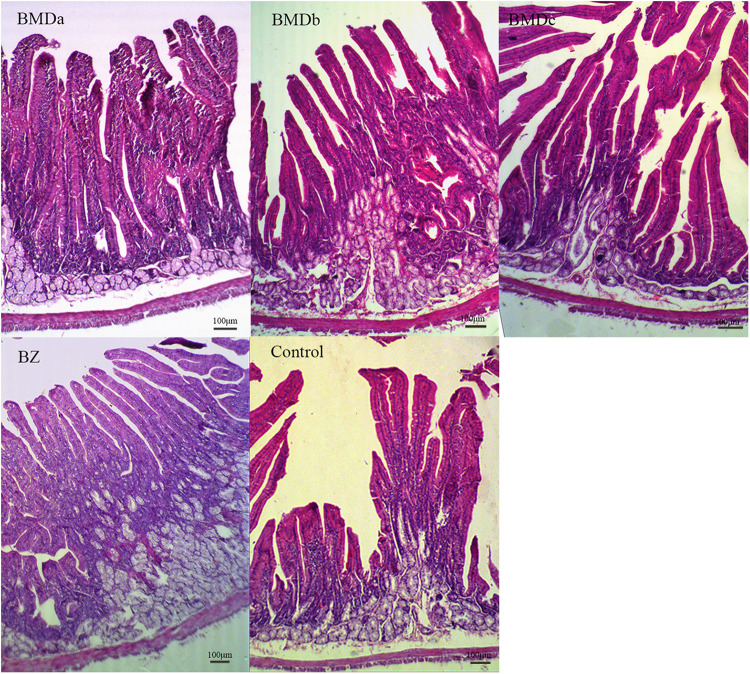
Effect of BMD on the morphology of duodenum in weaned rabbits.

As shown in Figure 1 and Table 1, duodenal histological analysis revealed that the villi were significantly longer in the BMDa, BMDb, and BZ groups than in the BMDc and control groups (*P* < 0.05). The crypt depth was significantly smaller in the BZ group than in other groups (*P* < 0.05), while it was not significantly different in the BMD groups and control group (*P* > 0.05). The villus/crypt ratio was significantly larger in the BMDa group than in the control group (*P* < 0.05). The duodenal wall thickness was significantly greater in the BMD and BZ groups than in the control group (*P* < 0.05). These findings indicated that BMD could affect the morphology to improve the absorption capacity of the intestine.

**TABLE 1:** Comparison of BMD based on the morphology of the duodenum in weaned rabbits.

Item	BMDa	BMDb	BMDc	BZ	Control
Villus (mm)	0.83 ± 0.057^a^	0.82 ± 0.088^a,b^	0.76 ± 0.053^b,c^	0.80 ± 0.037^a,b^	0.73 ± 0.043^c^
Crypt (mm)	0.27 ± 0.180^a^	0.27 ± 0.025^a^	0.30 ± 0.530^a^	0.19 ± 0.038^b^	0.32 ± 0.040^a^
Wall (mm)	0.06 ± 0.0054^a^	0.054 ± 0.011^a^	0.057 ± 0.012^a^	0.063 ± 0.013^a^	0.043 ± 0.0063^b^
Villus/Crypt	3.42 ± 0.440^b^	3.04 ± 0.410^b,c^	2.63 ± 0.570^b,c^	4.21 ± 0.420^a^	2.30 ± 0.400^c,d^

**Values are mean ± standard deviation (n = 28). ^*a*,*b*,*c*,*d*^ Indicate significant differences within a row (P < 0.05).**

### Intestinal Environment in Weaned Rabbits

As shown in Table 2, the concentration of acetic acid in the cecal contents was significantly higher in the BMD and BZ groups than in the control group due to BMD supplementation (*P* < 0.05). BMD, BZ, and control groups had no significant difference in propionic acid concentration, butyric acid concentration, and acetic acid/(propionic acid + butyric acid) (*P* > 0.05). In the cecal contents, the pH value was significantly lower in the BMDb group than in the other groups (*P* < 0.05); no significant difference was found in the pH values of the other groups (*P* > 0.05). The NH_3_-N content decreased gradually with the increase in BMD addition and was significantly lower in the BMD group than in the control group (*P* < 0.05). The NH_3_-N content was significantly lower in the BMDa group than in the BMDb and BMDc groups (*P* < 0.05), and significantly lower in the BZ group compared with the BMDc and control groups (*P* < 0.05). Therefore, supplementation with 50–100 mg/kg BMD increased the environmental acidity of the cecum in the weaned rabbits and reduced the NH_3_-N content to benefit the survival of useful bacteria in the intestines.

**TABLE 2:** Comparison of BMD based on cecal fermentation in weaned rabbits.

Item	BMDa	BMDb	BMDc	BZ	Control
Acetic acid (mg/mL)	0.95 ± 0.0220^a^	0.96 ± 0.0091^a^	0.87 ± 0.0098^a,b^	0.97 ± 0.0140^a^	0.80 ± 0.0079^b^
Propionic acid (mg/mL)	0.061 ± 0.0018	0.061 ± 0.0012	0.059 ± 0.0013	0.067 ± 0.0073	0.065 ± 0.0061
Butyric acid (mg/mL)	0.27 ± 0.0075	0.28 ± 0.0081	0.25 ± 0.0038	0.27 ± 0.0082	0.22 ± 0.0081
Acetic acid/(propionic acid + butyric acid)	2.87 ± 0.72	2.82 ± 0.66	2.81 ± 0.79	2.88 ± 0.84	2.80 ± 0.51
pH	6.68 ± 0.17^a^	6.36 ± 0.18^b^	6.63 ± 0.17^a^	6.74 ± 0.16^a^	6.60 ± 0.16^a^
NH_3_-N (mg/dL)	21.77 ± 3.12^d^	26.77 ± 3.80^b,c^	29.27 ± 4.29^b^	23.40 ± 3.76^c,d^	31.29 ± 4.51^a^

**Values are mean ± standard deviation (n = 28). ^*a,b,c*^ Indicate significant differences within a row (P < 0.05).**

### Gene Expression of Intestinal PGRPs in Weaned Rabbits

**FIGURE 2:**
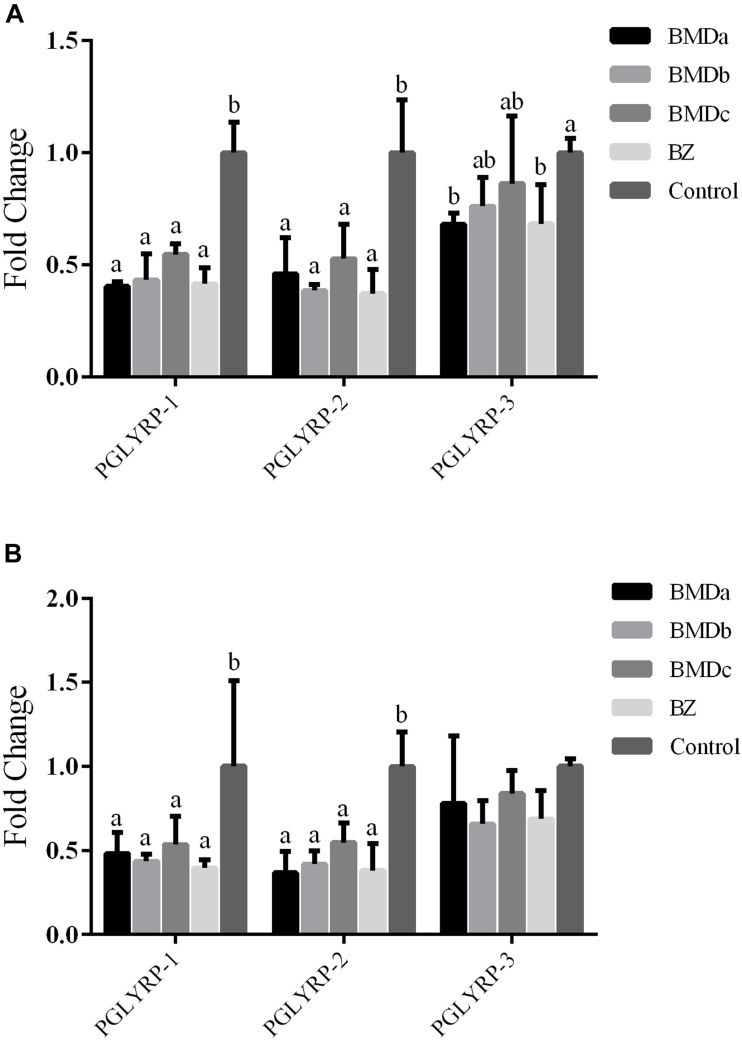
Expression of PGRPs in the cecum and duodenum. **(A)** Expression of PGRPs in the cecum. **(B)** Expression of PGRPs in the duodenum. a,b indicate significant differences within a row (*P* < 0.05).

The expression levels of *PGRPs* in different dose groups were tested to further verify the beneficial effects of BMD on the intestinal flora. The expression levels of *PGLYRP-1*, *PGLYRP-2*, and *PGLYRP-3* in the duodenum and cecum were detected. The expression levels of *PGRPs* in the duodenum and cecum followed the same trend, that is, the BMD and BZ groups had decreased expression levels compared with the control group (Figure 2). The expression levels of *PGLYRP-1* and *PGLYRP-2* were significantly lower in the BMD and BZ groups than in the control group (*P* < 0.05). The expression level of *PGLYRP-3* was not significantly different among BMD, BZ, and control groups (*P* > 0.05).

### Diversity, Richness, and Composition of Bacterial Communities in the Cecal Content

In terms of alpha diversity (Table 3), no differences in observed species, Shannon and Simpson indices, were noted in the BMDa, BMDb, BZ, and control groups. BMD and BZ supplementation increased the ACE and Chao1 richness compared with the control group, especially in the BMDa and BZ groups (*P* < 0.05). The NMDS analysis based on OTUs was conducted for comparison between the groups (Supplementary Figure 1). The stress was less than 0.2, indicating that the NMDS analysis had a certain reliability. The samples in these groups clustered due to different treatments. The findings combined with the MRPP analysis results showed that the differences in the microbial community structure among the groups were highly significant (*P* < 0.05), except the BMDa vs. BZ groups (Supplementary Figure 1 and Supplementary Table 3; *P* > 0.05).

**TABLE 3:** Number of observed species, richness, and diversity indices in the cecal content of each treatment.

Item	Experimental treatments				
	BMDa	BMDb	BMDc	BZ	Control
Observed species	1060.00 ± 90.09^a^	1017.25 ± 49.44^a^	957.25 ± 73.70^b^	1061.13 ± 96.90^a^	987.63 ± 92.95^a^
Shannon	7.4525 ± 0.3138^a^	7.4004 ± 0.4025^a^	6.4376 ± 0.6685^b^	7.5976 ± 0.4755^a^	7.2740 ± 0.6178^a^
Simpson	0.9730 ± 0.0061^a^	0.9719 ± 0.0088^a^	0.9235 ± 0.0458^b^	0.9756 ± 0.0123^a^	0.9650 ± 0.0174^a^
Chao1	1235.69 ± 108.76^a^	1141.81 ± 59.56^a,b^	1138.43 ± 75.46^a,b^	1231.40 ± 134.00^a^	1121.89 ± 135.38^b^
ACE	1222.52 ± 93.58^a^	1145.95 ± 54.21^a,b^	1137.07 ± 66.33^a,b^	1216.34 ± 109.72^a^	1111.47 ± 115.75^b^

**^*a,b,c*^ Indicate significant differences within a row (P < 0.05).**

**FIGURE 3:**
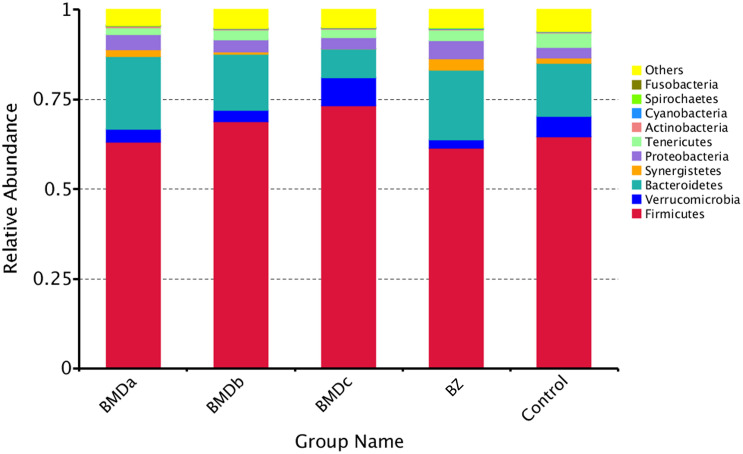
Percentage composition of the top 10 predominant phyla in the cecal content.

All the reads of the samples in the cecum were processed together. In total, bacterial sequencing generated 2,324,160 raw reads. Based on the > 97% sequence similarity, 2010 OTUs were identified, all of which belonged to the bacterial domain by the Greengenes classification. Moreover, 1,154 ± 91 OTUs per sample, on average, were identified. Within the bacterial population, the top 10 predominant phyla in the cecal content were identified across all samples at the class level (Figure 3). Firmicutes, Bacteroidetes, Verrucomicrobia, and Proteobacteria were the four dominant phyla, representing 66.29 ± 4.77%, 15.65 ± 4.89%, 4.50 ± 2.14%, and 3.74 ± 0.85% of the total sequences, respectively. Synergistetes and Tenericutes represented average percentages of 1.41 ± 1.19% and 2.77 ± 0.75%, respectively, of the total sequences. The proportion of phyla Actinobacteria, Cyanobacteria, Spirochaetes, and Fusobacteria was less than 1% of the total microbial community. Besides, the abundance of Firmicutes, Bacteroidetes, Proteobacteria, and Synergistetes presented a correlated change trend with the addition of BMD. In this aspect, the proportion of these phyla in the BMDa group most closely matched with that in the BZ group, followed by BMDb and control groups. Comparing the BMDa and BZ (positive control) groups with the control group revealed that the proportion of Bacteroidia, Gammaproteobacteriac, Deltaproteobacteria, Betaproteobacteria, and Bacillus increased while the proportion of Verrucomicrobiae and Mollicutes decreased due to the addition of high-dose BMD and BZ.

### Effect of BMD Supplementation on the Relative Abundance of Bacterial Communities

**FIGURE 4:**
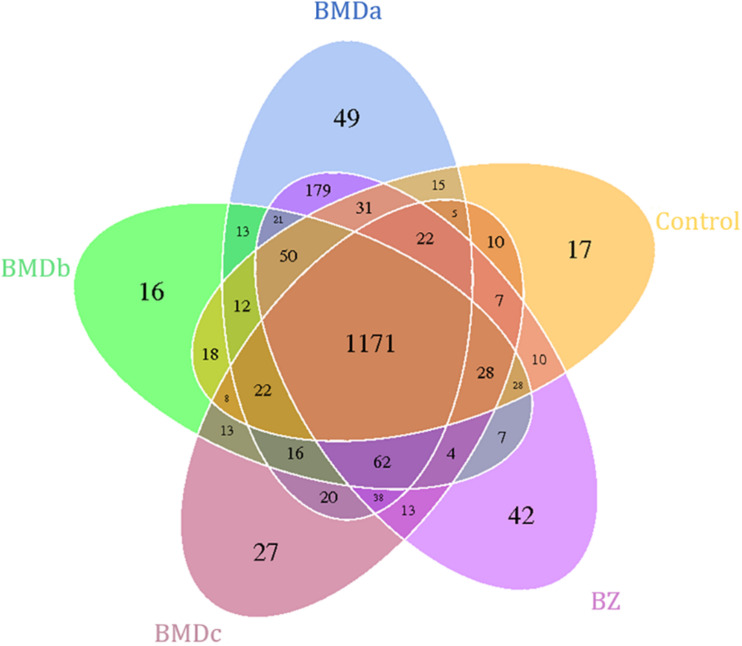
Shared operational taxonomic unit (OTU) analysis of the different groups. Each circle in the figure indicates a group, and the numbers in the circle and circle overlap represent the number of OTUs between the groups. The number not in overlap indicates the number of unique OTUs in the group.

The similarities and differences between the communities in different groups were compared and presented as a Venn diagram (Figure 4). The BMDa, BMDb, BMDc, BZ, and control groups had 1171 OTUs in common, with common OTUs comprising 67.84, 78.64, 79.88, 68.36, and 80.54% of the sequences in these communities, respectively.

The effect of BMD supplementation on the relative abundance of bacterial genera in the cecal content using *16S rRNA* sequencing is illustrated in Table 4. At the genus level, 113 taxa were detected, and 30 genera were affected (*P* < 0.05) by treatments. Compared with the control group, BMD and BZ supplementation increased (*P* < 0.05) the proportions of *Turicibacter, Epulopiscium*, and others. Specifically, the abundance of *Lachnospira*, *Erysipelotrichaceae* (*p-75-a5*), *Paraprevotellaceae* (*YRC22*), *Mogibacterium*, *Peptococcaceae* (*rc4-4*), *Anaerovibrio*, *Succinivibrio*, and *Sphaerochaeta* increased in the BMDa and BZ groups (*P* < 0.05). The relative abundance of *Alistipes*, *Sedimentibacter*, and *Dorea* significantly increased only in the BMDa group (*P* < 0.05).

**TABLE 4:** Effect of BMD supplementation on the relative abundance of bacterial genera in the cecal content detected using *16S rRNA* sequencing.

Phylum	Class	Order	Family	Genus	BMDa	BMDb	BMDc	BZ	Control
Euryarchaeota	Methanobacteria	Methanobacteriales	Methanobacteriaceae	*Methanobrevibacter*	6.32E-05 ± 6.99E-09^a^	4.97E-05 ± 3.71E-09^ab^	4.52E-06 ± 1.63E-10^b^	0.000158 ± 4.25E-08^a^	0.000325 ± 1.28E-07^a^
Bacteroidetes	Bacteroidia	Bacteroidales	[Odoribacteraceae]	*Butyricimonas*	0.000971 ± 5.45E-07^a^	0.0014 ± 3.76E-06^a^	0.0003071 ± 8.54E-08^b^	0.001449 ± 1.66E-06^a^	0.000777 ± 3.59E-07^ab^
Bacteroidetes	Bacteroidia	Bacteroidales	[Paraprevotellaceae]	*[Prevotella]*	0.000479 ± 1.83E-06^a^	4.52E-06 ± 1.63E-10^ab^	0^b^	0.000452 ± 1.63E-06^a^	9.03E-06 ± 6.53E-10^ab^
Bacteroidetes	Bacteroidia	Bacteroidales	[Paraprevotellaceae]	*YRC22*	0.000181 ± 2.61E-07^a^	0^b^	0^b^	0.000144 ± 1.55E-07^a^	0^b^
Bacteroidetes	Bacteroidia	Bacteroidales	Bacteroidaceae	*Bacteroides*	0.031261 ± 0.000366^a^	0.016744 ± 0.000138^a^	0.0097175 ± 5.13E-05^b^	0.023169 ± 0.000140529^a^	0.012901 ± 7.40E-05^a^
Bacteroidetes	Bacteroidia	Bacteroidales	Rikenellaceae	*AF12*	0.001386 ± 2.02E-06^a^	7.51E-07 ± 0.000199^c^	0.0001987 ± 2.13E-08^b^	0.001048 ± 2.32E-06^ab^	0.00042 ± 5.45E-07^ab^
Bacteroidetes	Bacteroidia	Bacteroidales	Rikenellaceae	*Alistipes*	8.81E-04 ± 6.41E-08^a^	0.000655 ± 7.85E-08^ab^	4.33E-04 ± 4.99E-08^b^	7.68E-04 ± 1.42E-07^ab^	4.56E-04 ± 3.80E-08^b^
Bacteroidetes	Bacteroidia	Bacteroidales	Rikenellaceae	*Rikenella*	0.002786 ± 7.51E-06^ab^	0.001662 ± 2.18E-06^ab^	0.0006819 ± 2.98E-07^b^	0.003378 ± 8.05E-06^a^	0.002917 ± 4.10E-06^a^
Firmicutes	Bacilli	Lactobacillales	Streptococcaceae	*Lactococcus*	0.000194 ± 3.02E-07^a^	1.81E-05 ± 1.12E-09^ab^	0^b^	0.00019 ± 2.88E-07^a^	1.35E-05 ± 3.50E-10^ab^
Firmicutes	Bacilli	Turicibacterales	Turicibacteraceae	*Turicibacter*	0.000235 ± 2.71E-07^a^	9.03E-05 ± 2.61E-09^a^	8.58E-05 ± 1.47E-09^a^	0.000217 ± 3.09E-07^a^	1.35E-05 ± 7.22E-10^b^
Firmicutes	Clostridia	Clostridiales	[Mogibacteriaceae]	*Mogibacterium*	6.77E-05 ± 3.67E-08^a^	0^b^	0^b^	5.42E-05 ± 1.94E-08^a^	0^b^
Firmicutes	Clostridia	Clostridiales	[Tissierellaceae]	*Sedimentibacter*	3.61E-05 ± 7.83E-09^a^	4.52E-06 ± 1.63E-10^ab^	4.516E-06 ± 1.63E-10^ab^	1.35E-05 ± 1.47E-09^ab^	0^b^
Firmicutes	Clostridia	Clostridiales	Lachnospiraceae	*Dorea*	0.000786 ± 4.41E-07^a^	0.000294 ± 7.25E-09^ab^	0.0003116 ± 3.71E-09^ab^	0.000786 ± 8.22E-07^ab^	0.000181 ± 8.20E-09^b^
Firmicutes	Clostridia	Clostridiales	Lachnospiraceae	*Epulopiscium*	0.003685 ± 6.15E-05^a^	0.000935 ± 7.62E-08^a^	0.0008399 ± 1.18E-08^a^	0.002 ± 3.10E-05^a^	9.03E-06 ± 2.80E-10^b^
Firmicutes	Clostridia	Clostridiales	Lachnospiraceae	*Lachnospira*	4.52E-05 ± 1.63E-08^a^	0^b^	0^b^	7.22E-05 ± 4.18E-08^a^	0^b^
Firmicutes	Clostridia	Clostridiales	Peptococcaceae	*rc4-4*	4.52E-05 ± 5.50E-09^a^	1.35E-05 ± 7.22E-10^ab^	9.031E-06 ± 2.80E-10^ab^	4.06E-05 ± 6.50E-09^a^	0^b^
Firmicutes	Clostridia	Clostridiales	Ruminococcaceae	*Anaerotruncus*	0.000881 ± 2.37E-06^ab^	0.000632 ± 6.26E-07^ab^	0.0004877 ± 1.38E-07^b^	0.001404 ± 1.66E-06^a^	0.001273 ± 1.01E-06^ab^
Firmicutes	Clostridia	Clostridiales	Ruminococcaceae	*Oscillospira*	0.032133 ± 3.36E-05^a^	0.031907 ± 1.39E-05^a^	0.0222708 ± 4.17E-05^b^	0.033785 ± 3.76E-05^a^	0.030498 ± 5.04E-05^ab^
Firmicutes	Clostridia	Clostridiales	Veillonellaceae	*Anaerovibrio*	0.000289 ± 6.22E-07^a^	0^b^	0^b^	0.000321 ± 8.22E-07^a^	0^b^
Firmicutes	Clostridia	Clostridiales	Veillonellaceae	*Veillonella*	0^b^	0^b^	0.001147 ± 1.05E-05^a^	0^b^	0^b^
Firmicutes	Erysipelotrichi	Erysipelotrichales	Erysipelotrichaceae	*[Eubacterium]*	1.35E-05 ± 7.22E-10^ab^	3.61E-05 ± 2.61E-09^a^	0^b^	1.81E-05 ± 1.49E-09^ab^	4.52E-06 ± 1.63E-10^ab^
Firmicutes	Erysipelotrichi	Erysipelotrichales	Erysipelotrichaceae	*Holdemania*	0.000122 ± 1.79E-08^ab^	0.000122 ± 8.55E-09^b^	0.0002167 ± 8.24E-08^ab^	0.000303 ± 1.45E-08^a^	0.000294 ± 2.03E-08^ab^
Firmicutes	Erysipelotrichi	Erysipelotrichales	Erysipelotrichaceae	*p-75-a5*	0.000113 ± 1.02E-07^a^	0^b^	0^b^	0.000163 ± 2.11E-07^a^	0^b^
Proteobacteria	Betaproteobacteria	Burkholderiales	Alcaligenaceae	*Sutterella*	3.61E-05 ± 7.83E-09^a^	0^b^	0.0001129 ± 1.02E-07^a^	5.42E-05 ± 2.35E-08^a^	0^b^
Proteobacteria	Gammaproteobacteria	Aeromonadales	Succinivibrionaceae	*Succinivibrio*	4.97E-05 ± 1.97E-08^a^	0^b^	0^b^	4.97E-05 ± 1.97E-08^a^	0^b^
Proteobacteria	Gammaproteobacteria	Enterobacteriales	Enterobacteriaceae	*Escherichia*	0.007491 ± 0.000374^ab^	0.000221 ± 1.40E-08^b^	0.0072701 ± 0.00039394^ab^	0.015258 ± 0.001418779^a^	0.00075 ± 1.07E-07^ab^
Proteobacteria	Gammaproteobacteria	Pasteurellales	Pasteurellaceae	*Actinobacillus*	0^b^	0^b^	0.0019462 ± 3.03E-05^a^	4.52E-06 ± 1.631E-10^ab^	0^b^
Proteobacteria	Gammaproteobacteria	Pasteurellales	Pasteurellaceae	*Pasteurella*	0^b^	0^b^	0.0016075 ± 2.07E-05^a^	0^b^	0^b^
Proteobacteria	Gammaproteobacteria	Pseudomonadales	Moraxellaceae	*Moraxella*	0^b^	0^b^	0.0001987 ± 3.16E-07^a^	0^b^	0^b^
Spirochetes	Spirochetes	Sphaerochaetales	Sphaerochaetaceae	*Sphaerochaeta*	9.93E-05 ± 7.90E-08^a^	0^b^	0^b^	9.48E-05 ± 7.19E-08^a^	0^b^

## Discussion

The intestinal mucosa serves as a filter between the intestinal lumen environment and homeostasis. It not only regulates the passage of nutrients and molecules but also prevents the penetration of bacteria, toxins, and dietary antigens into the submucosal tissues or circulatory system of the body (Groschwitz and Hogan, 2009). An effective intestinal barrier contains many constituent elements, such as the mucus layer, immune effect factor, and microbiome.

Changes in the intestinal tissue morphology can reflect the health of animals and their ability to digest and absorb nutrients to a certain extent (DeLegge, 2008). Weaned rabbits were susceptible to weaning stress and other changes in conditions, resulting in changes in tissue structure, such as intestinal villous atrophy, increased crypt depth, and mucosal damage, reducing the ability to absorb nutrients. The addition of BMD significantly increased the thickness of the intestinal wall, and also had a significant effect on the villus length and the villus/crypt ratio. The villi were longer in the BMD treatment groups compared with the control group in turkeys and pigs, which was typically equated with excellent gut health and high absorptive efficiency (Sims et al., 2004; Song et al., 2011). Moreover, the villus length in the BMDa and BMDb groups increased, indicating that the number of intestinal epithelial cells and the absorptive efficiency increased. The villus/crypt ratio was larger in the BMD group than in the control group, indicating that the addition of BMD changed the morphological structure of the duodenum, which was beneficial to the absorption of nutrients and maintained the steady state of the intestine.

The cecum is vital in the digestion and absorption of nutrients in rabbit diets, especially crude fiber. An appropriate cecal internal environment helped synthesize microbial proteins and prevent rabbit intestinal inflammation (Gidenne and Bellier, 2000). The main relevant indicators of the cecal internal environment included pH, VFA, and NH_3_-N. In this study, the addition of BMD increased the content of acetic acid, and the content of VFA stimulated the growth of intestinal mucosa, reduced alkaline damage, and enabled beneficial microorganisms in the intestine to grow and reproduce in a suitable environment. The content of NH_3_-N was significantly lower in the BMD group than in the control group. The content of NH_3_-N showed a significantly decreasing trend with the increase in the amount of BMD added, suggesting that the ability of microorganisms to use NH_3_-N was enhanced to facilitate the synthesis of more bacterial proteins. In summary, the addition of BMD caused pH, VFA, and NH_3_-N to interact with each other, resulting in a decrease in the alkalinity of the intestinal environment, which helped maintain a healthy state of the rabbit intestine.

It has been reported that BMD can regulate the innate immune responses in broilers (Brennan et al., 2013). However, the effect of BMD on intestinal immunity of rabbits is unclear. PGRPs were known innate immune-activating molecules highly conserved in insects and mammals (Liu et al., 2001; Dziarski, 2004). Up to now, 19 kinds of PGRPs have been found in insects. They activated Toll and IMD signaling pathways, induced protein lyase expression and phagocytosis, and participated in the hydrolysis of peptidoglycan and immunity against infection (Werner et al., 2000). Mammals contain four kinds of PGRPs, named PGLYRP-1, PGLYRP-2, PGLYRP-3, and PGLYRP-4 (formerly known as PGRP-S, PGRP-L, PGRP-Iα, and PGRP-Iβ, respectively), all possessing very high antibacterial activity (Kang et al., 1998; Liu et al., 2001). In previous studies, the expression levels of *PGLYRP-1*, *PGLYRP-2*, and *PGLYRP-3* in the duodenum and cecum of rabbits with diarrhea increased, which were related to the increase in pathogenic bacteria in the intestines (Chen et al., 2017). After adding BMD, the expression levels of *PGLYRP-1*, *PGLYRP-2*, and *PGLYRP-3* reduced compared with those of the control group. Both BMD and PGRPs have antibacterial effects, and their antagonism may cause the downregulation of PGRPs. However, the regulation mechanism in animals is very complicated. A more in-depth study is needed to prove the relationship between BMD, intestinal harmful flora, and PGRPs.

Since colonization, intestinal flora has become an important part of human and animal life. During the growth of the organisms, the flora and host interact to form a complex ecosystem that constantly changes and is relatively stable for a long time. Studies have shown that the imbalances caused by the changes in the composition, ratio, and diversity of early intestinal flora in infants and young children might be a potential factor for the occurrence of diseases, such as allergies, obesity, IBD, and intestinal colic, and largely affect the growth and development of diseases in the future (Videhult et al., 2016). Among monogastric livestock, the relative volume of rabbit cecum is larger. Microbes digested food residues in the cecum, and the cecum provided suitable conditions for the activities of microorganisms. In this study, the abundance of Clostridia, Bacteroidia, Synergistia, Deltaproteobacteria, and Betaproteobacteria displayed a correlated change trend with the addition of BMD, suggesting that BMD affected the diversity, richness, and composition of bacterial communities in the intestinal contents of weaned rabbits. Further, the analysis of the relative abundance of bacterial communities revealed that the proportion of *Turicibacter, Epulopiscium, Lachnospira, Erysipelotrichaceae (p-75-a5), Paraprevotellaceae (YRC22), Mogibacterium, Peptococcaceae (rc4-4), Anaerovibrio, Succinivibrio, and Sphaerochaeta* increased due to the addition of BMD and BZ. *Turicibacter* participated in fermentation metabolism, and lactic acid was its main metabolite. The mixture of *Turicibacter* with Clostridia restored intestinal serotonin in mice to normal levels (Yano et al., 2015). The abundance of *Erysipelotrichaceae* positively correlated with carbohydrate digestion (Cox et al., 2013; Young et al., 2013; Bermingham et al., 2017). The genus *Sphaerochaeta* lacked the genes encoding the characteristic flagellar apparatus and, contrary to most other spirochetes, acquired many metabolic and fermentation genes from clostridia (Caro-Quintero et al., 2012). This helped rabbits metabolize carbohydrates after eating high-fiber and low-protein forage. Among the affected genera, the proportion of *Alistipes, Sedimentibacter*, *and Dorea* increased only under BMDa treatment. *Alistipes*, as an intestinal bacterium, could produce short-chain fatty acids, which helped in the fermentation of food without producing alcohol. Generally, it improves the metabolic state of the intestine, which is more conducive to the use of glucose for energy (Yu et al., 2019). *Dorea* is the main gas-producing bacteria in the human intestine, which uses carbohydrates to produce gas. The proportion of *Dorea* in IBD significantly reduces, suggesting *Lachnospiraceae* (*Dorea*) is important in maintaining intestinal health (Sokol et al., 2017). These results suggested that BMD significantly changed the bacterial microbiome of the cecal content to affect intestinal function.

## Data Availability Statement

The original contributions presented in the study are publicly available. This data can be found here: https://doi.org/10.6084/m9.figshare.12609920.

## Ethics Statement

The animal study was reviewed and approved by the Institutional Animal Care and Use Committee of Yangzhou University.

## Author Contributions

YC was responsible for the collection, analysis of results and wrote the manuscript. SH, JL, and BZ performed experiments. NY, TZ, SL, and SB prepared figures and tables. XW designed the study. All authors read and approved the final manuscript.

## Conflict of Interest

The authors declare that the research was conducted in the absence of any commercial or financial relationships that could be construed as a potential conflict of interest.

## Footnotes

1 http://ccb.jhu.edu/software/FLASH/

2 http://qiime.org/scripts/split_libraries_fastq.html

3 http://drive5.com/uparse/

4 http://sourceforge.net/projects/rdp-classifier/

5 http://greengenes.lbl.gov/cgi-bin/nph-index.cgi

## Supplementary Material

Supplementary Figure 1 Non-metric multidimensional scaling analysis based on OTUs. Each point in the graph indicates a sample, and the distance between the points indicates the degree of difference. The same group of samples is shown in the same color.

Supplementary Table 1 Ingredient composition and nutrient levels of the basal diets.

Supplementary Table 2 Primers used for real-time qRT-PCR.

Supplementary Table 3 Differential analysis among groups using MRPP. The smaller the observed-delta value, the smaller the difference within the group; the larger the expected-delta value, and the greater the difference among groups. An *A* value > 0 means that the difference among groups is greater than that within the group; otherwise, it means that the difference within the group is greater than that among groups. A *P* < 0.05 indicates a significant difference.

## Abbreviations

ACE: Abundance-based coverage estimator
BMD: Bacitracin methylene disalicylate
BZ: Bacitracin zinc
CTAB: Hexadecyl trimethyl ammonium bromide
GAPDH: Glyceraldehyde-3-phosphate dehydrogenase
IBD: Inflammatory bowel disease
OD: Optical density
OTUs: Operational taxonomic units
MRPP: Multiresponse permutation procedures
NMDS: Non-metric multidimensional scaling
NCBI: National center for biotechnology information
PGRP: Peptidoglycan recognition protein
QIIME: Quantitative Insights Into Microbial Ecology
RDP: Ribosomal database project
VFA: Volatile fatty acid.
