# Supplementary figures and images for: Bacitracin Methylene Disalicylate Improves Intestinal Health by Modulating Its Development and Microbiota in Weaned Rabbits
Source: Front Microbiol. 2021 Jun 25;12:579006. doi: 10.3389/fmicb.2021.579006 (PMC8267888; doi:10.3389/fmicb.2021.579006)

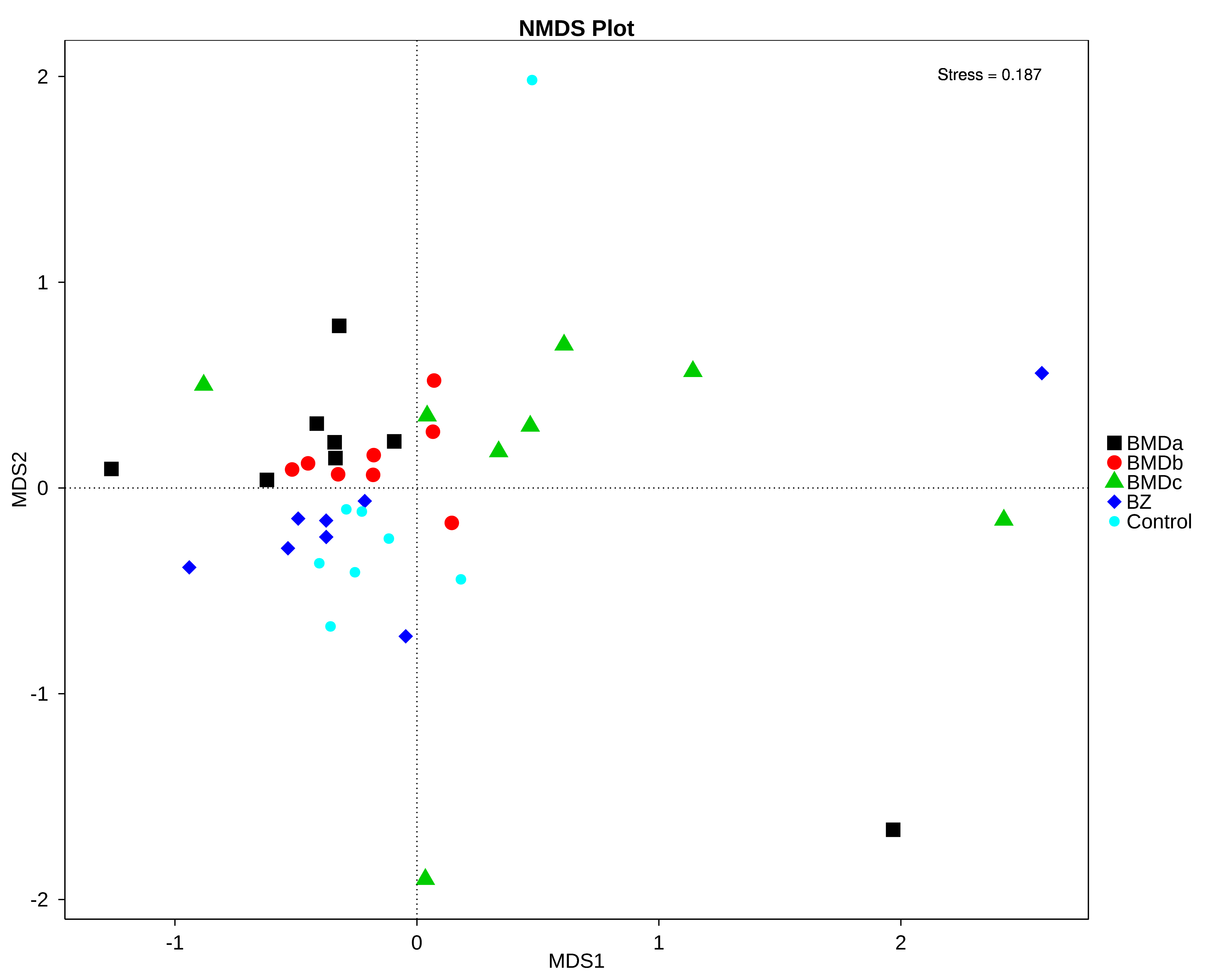

Supplement: Supplementary Figure 1 — Non-metric multidimensional scaling analysis based on OTUs. Each point in the graph indicates a sample, and the distance between the points indicates the degree of difference. The same group of samples is shown in the same color. [file Image_1.tif]
