# Supplementary material for: Bacitracin Methylene Disalicylate Improves Intestinal Health by Modulating Its Development and Microbiota in Weaned Rabbits
Source: Front Microbiol. 2021 Jun 25;12:579006. doi: 10.3389/fmicb.2021.579006 (PMC8267888; doi:10.3389/fmicb.2021.579006)
Supplement: Supplementary Table 1 — Ingredient composition and nutrient levels of the basal diets. [file Table_1.docx]

**Table S1.** Ingredient composition and nutrient levels of the basal diets

| Ingredient composition | Proportion (%) | Nutrient level | Proportion (%) |
| --- | --- | --- | --- |
| Alfalfa meal | 32.00 | Total energy (MJ/kg) | 14.25 |
| Corn | 23.00 | Crude protein (%) | 15.21 |
| Soybean meal | 13.00 | Crude fiber (%) | 14.71 |
| Bran | 22.00 |  |  |
| Oil bran | 8.00 |  |  |
| Dicalcium phosphate | 1.00 |  |  |
| Ore source | 0.50 |  |  |
| Salt | 0.50 |  |  |
