# Supplementary material for: Bacitracin Methylene Disalicylate Improves Intestinal Health by Modulating Its Development and Microbiota in Weaned Rabbits
Source: Front Microbiol. 2021 Jun 25;12:579006. doi: 10.3389/fmicb.2021.579006 (PMC8267888; doi:10.3389/fmicb.2021.579006)
Supplement: Supplementary Table 2 — Primers used for real-time qRT-PCR. [file Table_2.docx]

**Table S2.** Primers used for real-time qRT-PCR

| Name | Sequence (5′→3′) | Fragment size /bp |
| --- | --- | --- |
| GAPDH | F：GACTTCAACAGTGCCACC | 112 |
|  | R：TGCTGTAGCCAAATTCGT |  |
| PGLYRP-1 | F：CTGCTGCCTGCCGTGTGCGA | 115 |
|  | R：TCTGAAGACCCCAGCCCGGAG |  |
| PGLYRP-2 | F：ACTCCGTGGCTTCCTCCTG | 123 |
|  | R：TGCCCGTCCCGTACATCA |  |
| PGLYRP-3 | F：ATTTCGTAGAGCTGGTTGCC | 113 |
|  | R：CAGAGCCTGCTGGACTGC |  |
