# Supplementary material for: Bacitracin Methylene Disalicylate Improves Intestinal Health by Modulating Its Development and Microbiota in Weaned Rabbits
Source: Front Microbiol. 2021 Jun 25;12:579006. doi: 10.3389/fmicb.2021.579006 (PMC8267888; doi:10.3389/fmicb.2021.579006)
Supplement: Supplementary Table 3 — Differential analysis among groups using MRPP. The smaller the observed-delta value, the smaller the difference within the group; the larger the expected-delta value, and the greater the difference among groups. An A value > 0 means that the difference among groups is greater than that within the group; otherwise, it means that the difference within the group is greater than that among groups. A P < 0.05 indicates a significant difference. [file Table_3.docx]

**Table S3.** Difference analysis among groups using MRPP

| Group | *A* value | Observed-delta | Expected-delta | *P* value |
| --- | --- | --- | --- | --- |
| BMDa–BMDb | 0.01169 | 0.3569 | 0.3611 | 0.042 |
| BMDa–BMDc | 0.04834 | 0.4172 | 0.4383 | 0.001 |
| BMDa–BZ | 0.01517 | 0.3846 | 0.3905 | 0.147 |
| BMDa–Control | 0.05418 | 0.3754 | 0.3969 | 0.001 |
| BMDb–BMDc | 0.04201 | 0.3831 | 0.3999 | 0.002 |
| BMDb–BZ | 0.05057 | 0.3505 | 0.3692 | 0.001 |
| BMDb–Control | 0.0221 | 0.3414 | 0.3491 | 0.015 |
| BMDc–BZ | 0.07928 | 0.4108 | 0.4462 | 0.001 |
| BMDc–Control | 0.05491 | 0.4016 | 0.425 | 0.004 |
| Control–BZ | 0.0158 | 0.3691 | 0.375 | 0.01 |

The smaller the observe-delta value, the smaller the difference within the group; the larger the expected-delta value, the greater the difference among groups. An *A* value >0 means that the difference among groups is greater than that within the group; otherwise, it means that the difference within the group is greater than that among groups. A *P* value <0.05 indicates a significant difference.
